# Perspectives on incorporating expert feedback into model updates

**DOI:** 10.1016/j.patter.2023.100780

**Published:** 2023-07-14

**Authors:** Valerie Chen, Umang Bhatt, Hoda Heidari, Adrian Weller, Ameet Talwalkar

**Affiliations:** 1 Carnegie Mellon University, Pittsburgh, PA, USA; 2 University of Cambridge, Cambridge, UK; 3 The Alan Turing Institute, London, UK

## Abstract

Machine learning (ML) practitioners are increasingly tasked with developing models that are aligned with non-technical experts’ values and goals. However, there has been insufficient consideration of how practitioners should translate domain expertise into ML updates. In this review, we consider how to capture interactions between practitioners and experts systematically. We devise a taxonomy to match expert feedback types with practitioner updates. A practitioner may receive feedback from an expert at the observation or domain level and then convert this feedback into updates to the dataset, loss function, or parameter space. We review existing work from ML and human-computer interaction to describe this feedback-update taxonomy and highlight the insufficient consideration given to incorporating feedback from non-technical experts. We end with a set of open questions that naturally arise from our proposed taxonomy and subsequent survey.

## The bigger picture

Machine learning practitioners develop and deploy models that affect millions of people worldwide. Data scientists often receive feedback from domain experts like doctors, account executives, or regulators, all of whom may opine on a model and its outcomes. We ask: how can a data scientist be empowered to fix models to align better with the goals and values of experts? We provide an extensive review of methods for updating models based on expert feedback. For example, a radiologist could give feedback by drawing bounding boxes of where, in each X-ray, a specific fracture lies or by providing additional electronic health record data to co-reference a given scan. This feedback can then be used to augment the dataset to make a model more robust, alter the training objective to account for compliance objectives, or edit parameters to ensure that the model relies on the correct features. This review closes with concrete open questions to better involve non-technical experts in model development that are ripe for future work.

## Teaser

We review techniques that enable practitioners to incorporate feedback from domain experts into updates to machine learning models. Our work motivates the development of new methods for soliciting and leveraging feedback from experts.

## Introduction

**Figure 1:**
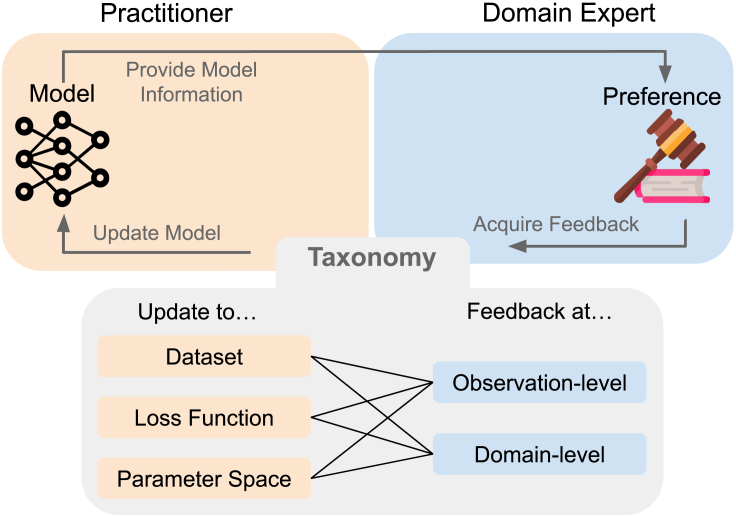
To incorporate an expert’s preferences to improve a model, practitioners must turn non-technical, domain expert preferences into usable model updates In this work, we propose a feedback-update taxonomy that focuses on the ways in which expert feedback can be translated into model updates. Our taxonomy has two axes, expert feedback and model updates, forming six categories of feedback-update interaction. We map existing work onto each category and use our taxonomy to motivate improving the interaction between practitioners and domain experts.

Before deploying a machine learning (ML) model in high-stakes use cases, practitioners, who are responsible for developing and maintaining models, may solicit and incorporate feedback from experts.^1^^,^^2^^,^^3^ Prior work has largely focused on incorporating the feedback of technical experts (herein, ML engineers, data scientists, etc.) into models.^4^^,^^5^^,^^6^^,^^7^^,^^8^^,^^9^^,^^10^ The feedback of technical experts might be immediately actionable, as likely few communication barriers exist between technical experts and practitioners. In contrast, the relationship between a practitioner and a non-technical expert (herein, doctors, lawyers, elected officials, policymakers, social workers, etc.), as illustrated in Figure 1, is more complex.^11^^,^^12^ Upon seeing information about the model, the expert provides feedback based on their preference to practitioners, who can then update the model. There has been insufficient consideration of how to incorporate feedback from non-technical, domain experts^13^^,^^14^ into models.

To bridge this gap, we start by examining model updates available to the practitioner and the types of feedback that non-technical experts might provide. We clarify the mechanisms available to turn feedback into updates and then devise a taxonomy along two axes: (1) levels of expert feedback and (2) types of model updates. Along the first axis (levels of domain expert feedback), expert feedback may come as domain-level feedback, which captures high-level conceptual feedback that the practitioner must translate into updates, or observation-level feedback, which captures how the model should behave on a few, specific data points.^15^^,^^16^^,^^17^^,^^18^ Along the other axis (types of model updates), we consider the updates a practitioner can make to a supervised learning objective, where feedback typically changes the dataset, the loss function, or the parameter space. (Supervised learning covers a broad range of model classes, ranging from vision transformers, large language models, and impactful application areas, like medical diagnostics^19^ and criminal justice.^20^ We consider other objectives, which may include reinforcement learning^2^^,^^21^ or unsupervised learning,^22^^,^^23^ to be out of scope for this review. We focus on methods more commonly deployed in practice and omit the Bayesian analog for our pipeline, where experts can express preferences over the distribution of functions.^24^^,^^25^) The two axes of our taxonomy form six distinct categories for feedback-update interactions. We place existing techniques from human-computer interaction, ML, robotics, and FATE (Fairness, Accountability, Transparency, Ethics) into each of these categories in mapping prior work to our taxonomy.

Our taxonomy not only provides a preliminary understanding on the ways in which non-technical expert feedback can be converted into practical model updates but also motivates a diverse set of open questions to improve practitioner-expert interactions. In open questions, we pose questions on how to connect model information to our taxonomy, how to prompt and elicit expert feedback effectively, and how to decide on the type of update to perform given the feedback. We hope that our taxonomy grounds the community in concrete ways to leverage non-technical expert feedback in a practical way while still encouraging future research to further feedback incorporation.

## Feedback-update taxonomy

One role of practitioners is to convert non-technical expert feedback into a model update. (We note the important case that, sometimes, valuable expert feedback might be received to say that using any model is not appropriate for the setting at hand. While such a concern must be taken seriously and considered carefully with relevant stakeholders, we do not discuss this case further here.) We describe the diverse ways that expert feedback can lead to model updates through our feedback-update taxonomy (Table 1). While experts are often involved prior to training an initial model,^19^ we focus on the iterative feedback process after a model has been trained. One piece of feedback could be used to alter multiple parts of the objective (e.g., change the dataset and loss function), but each update should be considered individually. We first elaborate on the two axes of our taxonomy and flesh out each category in the next section.

**Table 1:** Our feedback-update taxonomy illustrates the diverse ways practitioners can convert expert feedback, which generally comes via domain- or observation-level feedback, into model updates, which are either dataset, loss function, or parameter space changes that entail changes to the dataset, loss function, or parameter space, respectively

	Dataset update	Loss function update	Parameter space update
Domain feedback	dataset modification • augmentation^26^ • pre-processing^27^^,^^28^^,^^29^^,^^30^ data generation from constraint • fairness^31^ • interpretability^32^^,^^33^ weak supervision • using unlabeled data^34^^,^^35^^,^^36^ • checking synthetic data^37^	constraint specification • fairness^38^^,^^39^^,^^40^^,^^41^ • interpretability^42^^,^^43^^,^^44^^,^^45^ • resource constraints^46^^,^^47^	model editing • rules^48^ • weights^49^ model selection • prior update^50^ • complexity^51^^,^^52^
Observation feedback	active data collection • adding data^53^^,^^54^^,^^55^^,^^56^ • relabeling data^57^ • reweighting data^58^^,^^59^^,^^60^ • collecting expert labels^61^ passive observation^18^^,^^19^^,^^62^	constraint elicitation • metric learning^63^^,^^64^^,^^65^^,^^66^ • human representations^67^^,^^68^ collecting contextual information • generative factors^69^ • concept representations^70^^,^^71^ • explanations^72^ • feature attributions^73^^,^^74^	feature modification • add/remove features^75^^,^^76^^,^^77^ • engineering features^78^

Each cell corresponds to a subsection of mapping prior work to our taxonomy.

### Levels of domain expert feedback

Once an expert has observed information about the model, practitioners may ask for feedback to improve the model’s behavior in two general ways.^17^^,^^79^

• Domain feedback: it may be natural for non-technical experts to provide high-level conceptual feedback.^17^ The expert could provide explicit feedback over a set of good models^80^^,^^81^ or suggest data pre-processing to reduce discrimination.^27^^,^^28^^,^^29^
• Observation feedback: it may also be possible to learn by observing expert behavior.^15^^,^^16^^,^^18^ For example, practitioners can use observations to approximate a property of interest (e.g., fairness)^40^ or can collect contextual information, where every data point is accompanied by auxiliary information that can be used during learning (e.g., feature attributions,^74^ style factors,^69^ and semantically meaningful concepts^70^).

These two types of feedback form one axis of our feedback-update taxonomy in Table 1. While these two forms of feedback may be non-exhaustive, they capture a wide variety of mechanisms for non-technical experts to influence the development of models.^17^ Neither type requires non-technical experts to have knowledge about the model or the training process itself. A radiologist could provide domain-level feedback about X-ray scans via high-level information about the region of interest in each X-ray or pre-processing suggestions for every scan. Examples of observation-level feedback that the same radiologist could provide on X-ray scans include bounded boxes of where in each X-ray a specific fracture lies or additional electronic health record data to co-reference a given scan. The role of practitioners may be expanding, as a practitioner may need to decide, as part of the model update process, whether to treat the collected feedback as domain- or observation-level feedback.

We consider other forms of feedback to be out of scope for this work because they are less intuitive to elicit from a non-technical domain expert.^82^^,^^83^ This includes changing the learning algorithm (e.g., in differential privacy communities^9^^,^^84^), selecting hyperparameters (e.g., in AutoML research^5^^,^^85^), and specifying the order of data points given to a learning algorithm (e.g., in machine teaching literature^6^^,^^8^^,^^10^).

### Types of model updates

In the supervised learning setting, a practitioner generally minimizes a loss function on a provided dataset to learn the parameters of a model. Once experts have provided feedback, practitioners can leverage expert input to improve the model in multiple ways: updating the dataset, the loss function, or the parameter space. These update types form the other axis of Table 1.

• Dataset updates: feedback can be incorporated by changing the dataset on which the model is trained.
• Loss function updates: feedback can also be incorporated by adding a constraint to the optimization objective. This manifests as a change in the loss function.
• Parameter space updates: finally, feedback can also be provided on the parameters of the model itself, which reflect a change in the parameter space.

For example, a public official may ask that as the input feature population increases, the likelihood of a project proposal getting funded should increase; this implies monotonicity between an outcome and input feature. Practitioners can incorporate this feedback in various ways, which are different technically. The practitioner can update the dataset by adding or removing appropriate data points, update the loss function by adding a regularizer that penalizes the model for not satisfying this condition, or update the parameter space by optimizing over a subspace of parameters that satisfy this condition.

While these update types may seem straightforward, it is unclear how to identify the new dataset, loss function, or model parameter space: this involves transforming domain expert feedback into one of these three general updates. After the practitioner incorporates expert feedback, the updated model should ideally reflect the expert’s preferences better than the original model. In the next section, we flesh out the conversion from feedback to update.

## Mapping prior work to our taxonomy

For each feedback-update pair, we illustrate how the practitioner can use the feedback to modify the ML model. We provide key takeaways for each feedback-update pair, summarizing current work and future directions. To identify relevant work in each category of the taxonomy, we used a snowball sampling methodology^86^ to gather references that pertain to expert feedback incorporation. For clarity, we provide examples for each category to ground our discussion. While each category is its own research direction, we provide a broad, non-exhaustive discussion of how to update models under expert feedback. Note that many references we provide do not consider a domain expert explicitly but can be applied to the feedback that the domain expert provides. While all feedback may not always be immediately actionable, we hope our paper encourages future work to help practitioners address non-technical experts’ needs and concerns more efficiently.

### Domain to dataset

**Figure 2:**
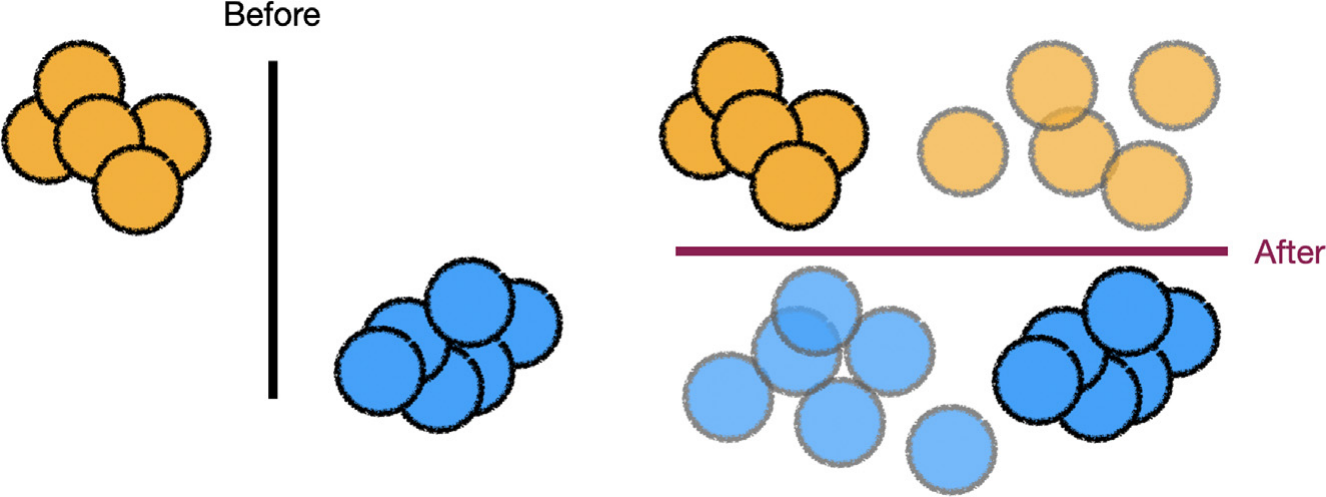
The practitioner generates data, shown in translucent circles, to remove reliance on one feature, after retraining, per the expert’s domain-level feedback The updated dataset is used to learn a new model.^36^

A dataset can be modified given domain-level feedback. The practitioner can modify the original data or generate new data. If the expert provides domain-level feedback that the model should not rely on a feature, then adding data can lead to a model that satisfies this expert-specified domain-level feedback, as shown in Figure 2.

#### Dataset modifications

A non-technical expert may specify feedback that entails a dataset modification and, consequently, a retraining of the model. One example that naturally suggests a dataset update is dataset pre-processing; here, a new perturbed dataset is obtained by applying a transformation to features of the original dataset. In many cases, the sizes of the new and old datasets are the same. Suppose the expert suggests that the dataset should be balanced in terms of the sensitive attribute; the practitioner can design a transformation function that achieves this property. Extensive work has been done on data pre-processing for fairness.^27^^,^^28^^,^^29^ Another example of domain-level feedback that leads to dataset updates is data augmentation, where an augmentation scheme is applied to every dataset point in the original dataset, yielding a dataset of original points and augmented points. In these settings, the size of the dataset tends to be larger after augmentation. After the expert specifies that the model should make the same prediction regardless of image rotation, the practitioner may consider modifying the dataset by adding rotated variants of input images to the dataset,^87^ which allow for more robust inference.^26^

#### Data generation

Other domain-level feedback may not necessarily suggest a dataset modification but rather may prescribe a way to generate data to augment the existing dataset. For example, an expert may say that the model should not rely on spurious correlations between people and other objects in images (e.g., using a tennis player to detect the tennis racket). A practitioner may generate counterfactual images from the original dataset that contain and do not contain the people and other objects to decrease reliance on the person in the image.^33^ Generative modeling techniques are useful to create synthetic data that adhere to a property specified by an expert.^88^ For example, to assuage an expert’s fairness concerns, a practitioner could generate data under a fairness constraint per Xu et al.^31^

#### Weak supervision

Domain-level feedback may be used to handle unlabeled data. Weak supervision applies to any approach that deals with data where only some is labeled. To leverage additional unlabeled data, we can obtain lower-quality labels efficiently.^89^ The most common approach is to ask experts to provide higher-level supervision over unlabeled data.^34^^,^^35^^,^^36^ For example, an expert may provide an explicit rule stating that “all individuals under 18 should have a negative label.” The practitioner can turn this rule into a pseudo-labeling function, which can be used to leverage large amounts of unlabeled data points.

##### Takeaway

Approaches to modifying or generating data are common in the ML literature. However, these methods do not usually involve eliciting supervision from non-technical experts. Future work should focus on prompting experts for domain-level feedback, which could induce dataset changes in settings where collecting more data may be hard: we elaborate on this in difficulties with feedback collection.

### Domain to loss function

**Figure 3:**
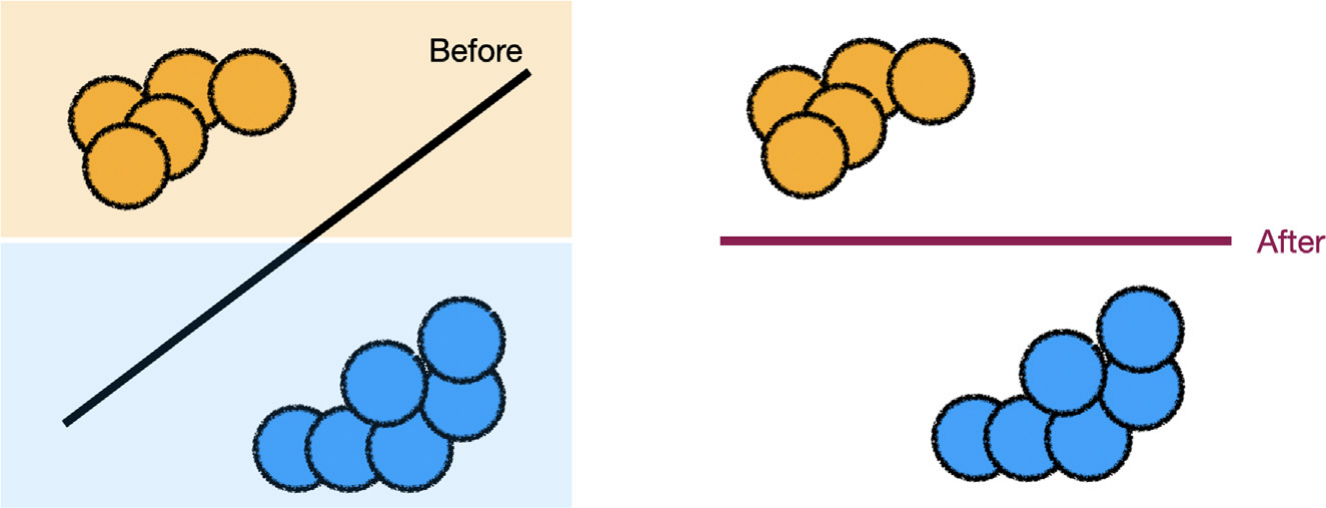
The expert specifies that model behavior should be similar in each color block The practitioner converts this feedback into a regularizer, which is used in the new loss function and obtains an updated model.^90^

Practitioners can use an expert’s domain-level feedback to update the loss function. One example is shown in Figure 3.

#### Constraint specification

Experts may have access to properties, beyond performance, that may be well suited to constrained optimization. There has been a diverse body of work on incorporating fairness^38^^,^^41^^,^^91^ and interpretability^42^^,^^43^ constraints. Other concerns experts may express include memory constraints that prohibit models with many parameters^46^ or run time constraints that require low-latency inference.^47^ For many of these constraints, practitioners may update their loss function to incorporate a regularization term. For example, to achieve better fairness outcomes with respect to demographic parity, Slack et al.^90^ added a regularizer that accounts for the accuracy of the protected instances. Other work improved interpretability by placing constraints on the neighborhood fidelity of the model at each point,^43^ which some experts may desire.

##### Takeaway

The literature demonstrates that it is possible to add property-like constraints to the loss functions. Prior work fails to ask non-technical experts to specify constraints for two potential reasons. First, providing model information that captures relevant property information may be more difficult to capture than information about specific data points. Second, specifying constraints in a usable (e.g., mathematical) format may be challenging for non-technical experts. For example, experts may desire properties that are difficult to convert into a precise statement that can be incorporated in the loss function (e.g., societal norms/ethics).

### Domain to parameter space

**Figure 4:**
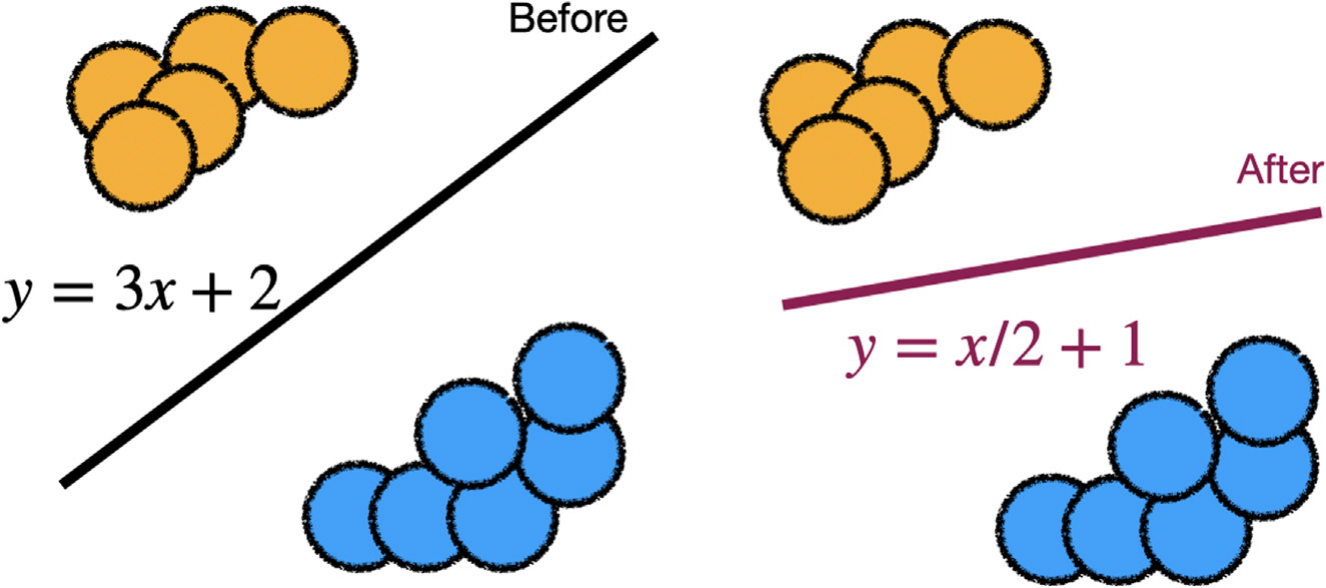
For certain types of models, the expert may be able to directly edit the model parameters Here, the expert changes the coefficients of the linear model to obtain their desired model.^49^

Specified domain-level feedback can reduce the parameter space to a set of potential models. In Figure 4, we show a simple setting where the expert can directly intervene on model weights.

#### Model selection

It may be possible to have the expert provide preferences over a set of models. Though there are a number of open questions about how this set is presented (discussed in difficulties with feedback collection), one solution from Lage et al.^50^ is to show an explanation to an expert, calculate the amount of time it takes for the expert to predict the label, and change the model prior (update) accordingly. The change to the model prior manifests as constraints the practitioner places during fine-tuning to ensure that the resulting model is interpretable given a specified tolerance. The model prior can also be implicitly changed by imposing complexity constraints on the set of possible models,^51^^,^^52^ which may include constraints on how sparse, interpretable, or smooth the model must be.

#### Model editing

For some model classes (e.g., simple, transparent models^48^^,^^49^), the expert may directly provide feedback that would change the parameters of the model itself without requiring retraining of the model on new data or loss function. For example, Wang et al.^49^ allow experts to directly change the weights of a generalized additive model (GAM) after exposing shape function visualizations and other model properties. In this case, the update to the parameter space is trivial because the set of possible parameters is the exact model that is specified by the expert. These edits on the model might implicitly align the model with unexpressed, desired expert properties, allowing practitioners to avoid sample-inefficient data collection procedures and potential difficulties with constraint specification.

##### Takeaway

While these types of updates traditionally require more technical users, there are increasingly more user-friendly interfaces developed to allow even non-technical experts to edit the model in a more direct manner.

### Observation to dataset

**Figure 5:**
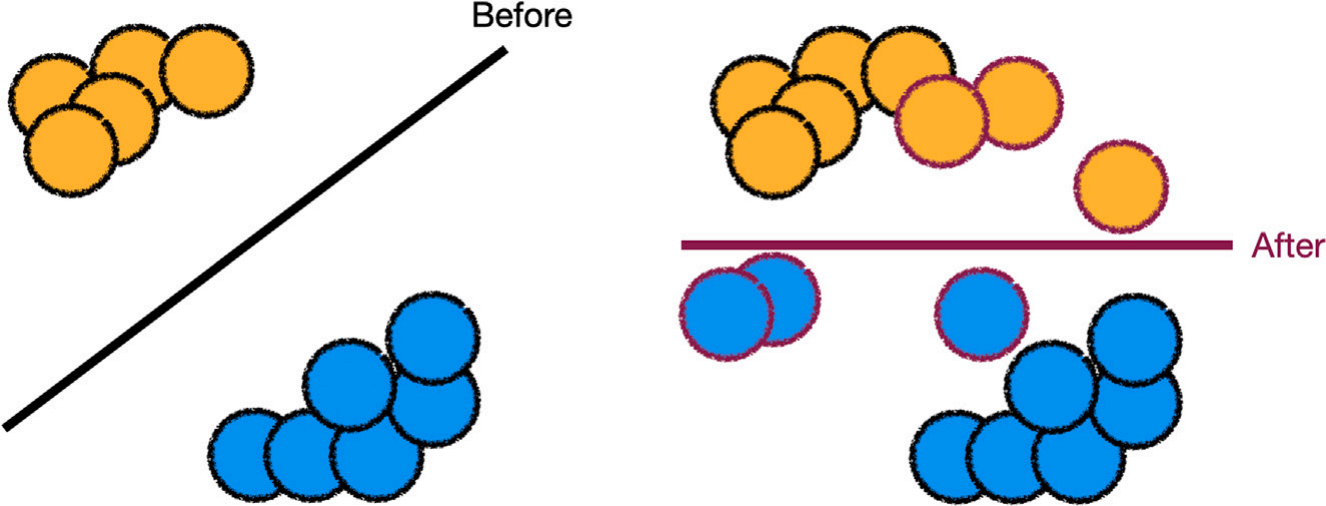
The stakeholder labels new points, which are denoted by the purple circles These points are then added to the dataset and used to obtain an updated model.^19^

The dataset can be directly modified from observation-level feedback via actively collecting data (e.g., asking experts to label selected data points) or passively observing behavior (e.g., collecting data from expert behavior in practice). In Figure 5, we show how collecting new data can transform the model.

#### Active data collection

The field of active learning falls into this category.^92^^,^^93^ Traditional work on active learning does not explicitly consider the human in the loop (i.e., the choice of new points to add to the dataset are selected by a learning algorithm), and it is rather straightforward to use the updated dataset to retrain the model. Data collection may involve asking the expert to approve the weight placed on a model^58^ or to provide a label for a data point.^57^ Experts may also review new data points, where each new data point is selected by some heuristic (e.g., high uncertainty regions) and the corresponding label is specified by the expert.^54^^,^^56^ Some work considers collecting multiple labels from various experts for each data point.^61^ Recently, active learning has been studied alongside model transparency, specifically using explanations to assist experts with choosing which points to add to the dataset.^55^ Cabrera et al.^53^ propose an extensive visual analytics system that allows experts to verify and produce examples of crowdsourced errors, which can be thought of as additional data.

#### Passive observation

Instead of asking the expert to provide labels on additional pieces of data, the practitioner could also collect data via expert demonstrations. Inverse decision theory argues for observing human decisions to learn their preference.^18^ One can simply observe a non-technical expert’s behavior to generate more data.^17^ For example, a data scientist might choose to wait for radiologists to see more patients before updating the dataset and model.^19^ While this may not be the most efficient way to perform model updates, Laidlaw and Russell^62^ find that forcing humans to make decisions under uncertainty can lead to better preference learning. Note that the online learning community has extensively studied how to incorporate expert knowledge into learning statistical models based on a sequence of observation.^94^^,^^95^^,^^96^

##### Takeaway

Active data collection may not immediately appear to be reasonable feedback to expect from non-technical experts. Compared with the traditional crowd workers used in ML literature, non-technical experts like lawyers and regulators may not have the time to extensively label data that are required for some powerful deep learning methods. However, we do not exclude this work because it may still be desirable to collect data from non-technical experts (e.g., in settings with limited amounts of data) or collect observation-level feedback in a passive manner.

### Observation to loss function

**Figure 6:**
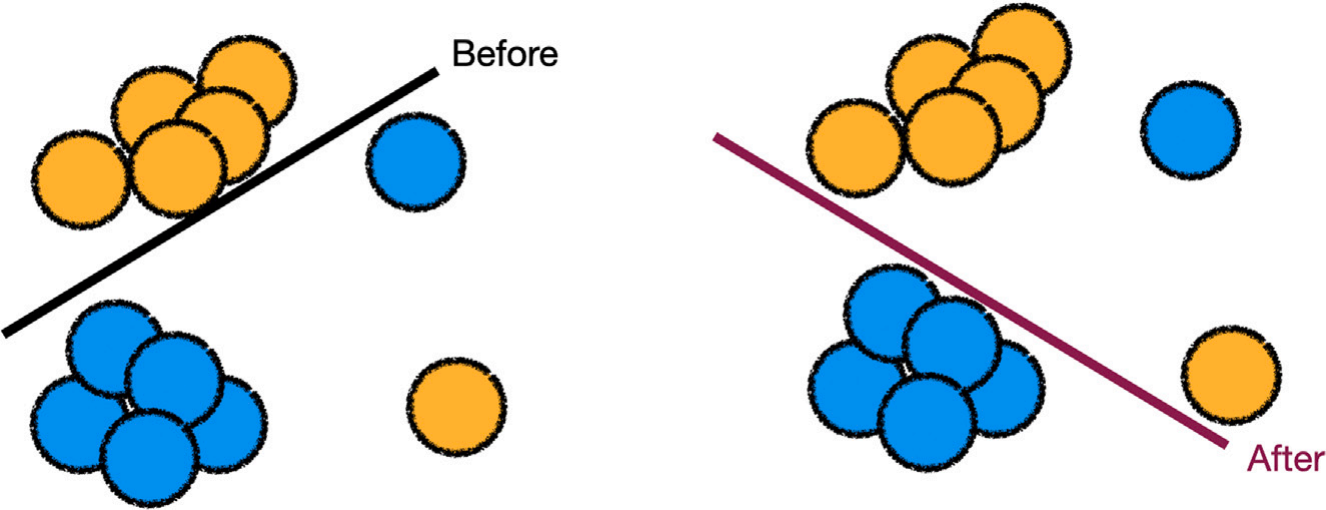
The expert specifies that the cost of mislabeling a yellow point is higher than the cost of mislabeling a blue point Retraining with a loss function that incorporates the cost of error yields a cost-sensitive model.^97^

We identify two ways to collect additional information to learn and integrate a new function into the loss. In Figure 6, we show that practitioners can collect contextual information in the form of error costs to edit the loss function and retrain the model.

#### Collecting contextual information

Experts may have contextual information they wish to share with practitioners. While there is not a unified way for practitioners to incorporate contextual information, it is often used for a loss function update. The contextual information might be provided for some subset of points if collecting it for all data points is expensive.^97^^,^^98^ In general, contextual information can be used to constrain the model behavior.^99^^,^^100^

Tseng et al.^73^ and Weinberger et al.^74^ both modify the loss function to regularize feature attributions to ensure that the explanations from models better align with expert expectations. Koh et al.^70^ proposed concept bottleneck models (CBMs) as a way to incorporate pre-defined concepts into a supervised learning procedure, where the concepts are semantically meaningful pieces of information used in a discriminative model to perform prediction.^101^^,^^102^ Their supervised approach maps raw inputs to concepts and then maps concepts to outputs. An intermediate layer of a neural network can also be selected as the CBM, where the layer’s activations should be aligned with concepts when training.^7^^,^^71^

Furthermore, expert-specified contextual information can be used in other clever ways. Vapnik et al.^103^ use privileged information for each input to accelerate learning of a support vector machine. Abe et al.^104^ use misclassification costs to find a weighted loss function that improves model performance under class imbalance. Adel et al.^69^ use generative factors to improve deep representation learning. Hind et al.^72^ use explanations to partition classes into subclasses for more accurate models downstream.

#### Constraint elicitation

The practitioner may choose to parameterize a constraint using observation-level feedback. Analogous to metric learning,^105^ this can be done by learning the hyperparameters of a constraint from expert feedback about individual points^66^ or by building a function from expert observations.^65^^,^^106^^,^^107^ The learned metric is then appended onto the existing loss function. For example, some have attempted to learn an individual fairness constraint after receiving pairwise judgements from experts, who specify if two individuals should be treated the same or not.^65^^,^^107^ Practitioners can also constrain intermediate model representations^67^ or edit a model’s representation,^68^ which entails experts selecting exemplar training points that should have similar representations to a test point.

##### Takeaway

The aforementioned approach of constraint specification (see domain to loss function) bears similarity to both approaches we discuss here. The main difference between using constraint specification and collecting contextual information is that contextual information is specified for each data point, which may be easier for an expert to provide generally. Although constraint elicitation techniques circumvent the potential difficulty of constraint specification, these techniques require more creativity in how the individual pieces of observation-level feedback can be combined. We note that there should be further work on organizing these types of approaches, as they likely will be domain specific.

### Observation to parameter space

**Figure 7:**
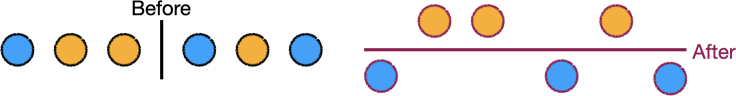
The expert adds a new feature to the dataset This addition allows the data points to become linearly separable, which leads to a more accurate model.^75^

In some cases, the parameter space may not be rich enough to find a suitable model that fits the dataset well.^62^ Additional data containing modified features can be used to alter parameter space. In Figure 7, we show how adding a feature to a dataset allows the updated model to better separate the blue and yellow points.

#### Feature modification

This type of update manifests as a change to the parametric form of the model, which now makes predictions on the changed data points. Correia and Lecue^76^ and Roe et al.^108^ use experts to select a subset of features to use for prediction (e.g., to bias the model away from using spurious correlations). Bakker et al.^75^ sequentially add features to a dataset to achieve fairness goals. In these works, the original parameter space may be all two-dimensional models, but the updated parameter space after feature acquisition would be three-dimensional. Moreover, there is a plethora of work on feature selection that implicates the selected model class.^109^^,^^110^ There have been works where experts can suggest the model class based on their interpretability needs: the less complex the parameter space, the more interpretable the model.^51^^,^^52^

##### Takeaway

Working with experts to modify features might be particularly helpful earlier in model development. We note that this type of update is already commonly done in practice.

## Open questions

**Figure 8:**
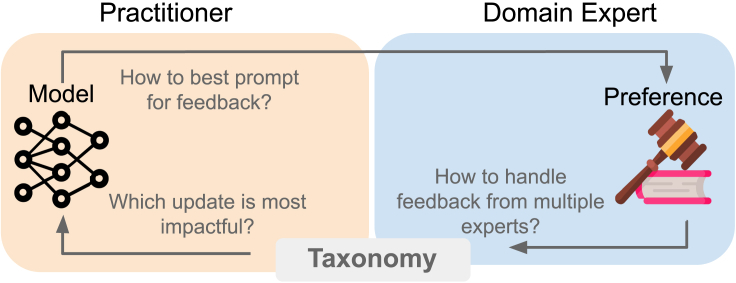
Beyond the feedback-update taxonomy introduced in mapping prior work to our taxonomy, we present open questions in the broader pipeline that a practitioner follows to solicit and incorporate feedback from stakeholders

Through our review of practitioner-expert interactions, we find there are multiple important and exciting open questions. While our taxonomy highlights many ways to convert feedback to updates, there are still open questions about the entire feedback solicitation and incorporation process, as illustrated in Figure 8. We discuss open questions of how practitioners should prompt and collect feedback from non-technical experts (difficulties with feedback collection) and how practitioners can decide the best type of update to perform given feedback (problems with updating models). We discuss the potential algorithmic and participatory innovations needed to improve our feedback-update pipeline: collaborative effort between multiple communities is imperative to increase non-technical expert involvement in model development.

### Difficulties with feedback collection

There are many complexities that arise when practitioners collect feedback from non-technical experts, which include *p*rompting for feedback and engaging with multiple experts.

#### Prompting for feedback

What information about the model should the practitioners present to the expert? What type of prompt should a practitioner choose?

In the process of prompting experts for feedback, practitioners need to decide how and what information about the model needs to be shown to experts. There has been initial work to develop interfaces to visualize trade-offs between multiple objectives,^111^ to allow experts to explore model behavior,^112^^,^^113^^,^^114^ or to interact with white-box proxies.^48^ There has been a flurry of ML monitoring work, which provides dashboards to assess models in production.^115^^,^^116^ While these techniques try to bridge the communication gap between practitioners and experts, these tools seldom provide adequate remediation for experts to express their preferences or for practitioners to incorporate those preferences.

**Figure 9:**
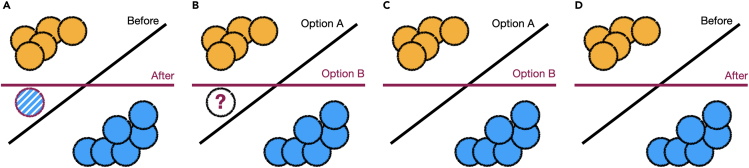
We illustrate four different combinations of feedback type and prompt styles that a practitioner might ask from an expert (A) The practitioner presents a model, and the expert specifies the addition of the striped point with a purple border to the blue class. The induced change by this feedback yields a new model. (B) The practitioner presents the expert with a forced choice to provide a label for the point denoted with a question mark. Depending on the selected label, the induced classifier may be option A or option B. (C) The practitioner presents the expert with a forced choice of two properties: enforce max-margin between two classes (option A) or avoid reliance on the vertical feature (option B). (D) The expert specifies a property that the model should not use the vertical feature to make predictions, inducing the same classifier (option B) in (B) and (C).

Given that experts might want to provide different types of feedback,^117^ an important open question is how practitioners should guide non-technical experts via prompts to provide specific feedback. Two such prompt styles include open-ended feedback and forced-choice feedback. In Figure 9, we differentiate how these feedback types can be presented with a simple example.

However, challenges arise both in dealing with the types of prompts^118^ and deciding how to word the prompt.

In open-ended feedback, experts are unconstrained in the information they can provide. Thus, the onus is on experts to identify relevant aspects of model behavior on which to opine and to do so in a usable manner.^119^ Open-ended feedback is more challenging to use insofar as the feedback may have little to no implication on the model. For example, subjective or qualitative feedback (e.g., measures of confidence or trust^120^^,^^121^) may be difficult to translate into downstream model updates.

Forced-choice feedback is feedback presented to the expert in the form of options, where the expert is forced to pick either one or multiple options or rank the options. There is a related question of how to select, visualize, and present options. For example, in the growing body of work that studies a *ε*-Rashomon set of good models,^122^ there are currently only heuristics to identify this set of models. This may be done by setting multiple random seeds,^81^ optimizing under constraints,^123^ or subsampling from the dataset^124^; nonetheless, it remains unclear how to visualize this set.

##### Algorithmic innovation

For open-ended feedback, new methods can let experts better traverse model behavior to improve holistic understanding of the model. For forced-choice feedback, future work can build methods to understand the order in which the queries should be asked and what set of options to provide.

##### Participatory innovation

The practitioner’s choice of content and presentation of model information will affect the expert’s downstream feedback.^125^ Additional user studies are needed to understand the most appropriate pairings of model transparency, prompts, and interfaces to guide the development of useful tools to involve non-technical experts, recognizing potential challenges.^126^^,^^127^^,^^128^

#### Engaging with multiple experts

How does a practitioner perform an update after receiving feedback from multiple experts?

When non-technical domain experts are considered in our pipeline, there may be multiple, diverse experts who want to express feedback on a model. Thus, it is important to expand our taxonomy to handle multiple domain experts. In an ideal world, there would be an easy way to incorporate feedback from multiple domain experts into models: this is related to participatory ML,^14^ a burgeoning community democratizing the ability for all to interact with models.

Practitioners should combine feedback from multiple domain experts carefully, perhaps using an aggregation mechanism.^129^^,^^130^ The combinatorial auction literature has long studied how to best elicit preferences when the number of options is combinatorial.^131^^,^^132^ Every feedback aggregation mechanism is value laden, as some mechanisms might prefer one expert over others and some tie-breaking mechanisms might under-represent some experts.^133^^,^^134^^,^^135^^,^^136^ Even if practitioners had a sensible feedback aggregation mechanism, settling incongruities in feedback is difficult, as there might be conflicting pieces of feedback from multiple, diverse domain experts.^119^^,^^129^^,^^130^^,^^137^^,^^138^

##### Algorithmic innovation

Developing methods to aggregate feedback from multiple domain experts will require a framework that not only considers the heterogeneous feedback types but also permits various aggregation schemes, which combine potentially contradictory feedback into a collective piece of feedback for the practitioner to incorporate.^139^^,^^140^

##### Participatory innovation

New methods should consider ways to efficiently elicit feedback at scale from diverse domain experts and mechanisms to make explicit the value-laden aggregation done when ironing out potential contradictions in collected feedback.

### Problems with updating models

There is a gamut of considerations when practitioners decide to update models based on expert feedback. We consider when to update based on feedback and which update to use.

When should a practitioner incorporate an expert’s feedback? How can a practitioner best collect feedback to make the most impactful update to the model?

Non-technical domain expert feedback is difficult to collect.^50^^,^^67^ As a result, practitioners need to ensure that, given a single piece of feedback, the update they make has a large impact on the model. To measure the impact of an update, there are a few open questions to address. First, it is not clear what impactful means. One naive way is to measure the amount of feedback needed to affect a specified change in the model. However, this ignores how different types of feedback may have different costs of collection and may be amenable to different types of updates.

In settings where feedback suggests that an expert solely cares about performance on a test distribution identical to the training distribution, the practitioner may not be able to make meaningful changes to the model beyond what is learned by the empirical risk minimizer.^41^ In some sense, collecting and incorporating expert feedback is only helpful when the expert cares about objectives beyond performance, which may include interpretability,^141^ robustness,^61^ or fairness.^107^ Even after collection, the feedback an expert provided may not always result in a meaningful change to the model. For example, in the late stages of training, additional supervision for in-distribution data or domain-level feedback, which has already been implicitly captured via a sufficiently large amount of labeled data, may prove fruitless.

A practitioner may need to choose between feedback-update pairs because multiple update types could be interchangeable given a piece of feedback. For example, a domain expert’s fairness goals can be achieved by clever sampling from the dataset^142^ or by adding constraints to the loss.^41^ Others have connected loss function and dataset changes using optimization,^143^ Bayesian methods,^144^ and group theory.^145^

#### Algorithmic innovation

There is much work to be done in comparing update types to understand what updates are easier to make for what feedback. Understanding the conditions under which practitioners can use either type interchangeably will be important. From a technical perspective, practitioners can estimate the complexity of an update (e.g., as in Laidlaw and Russell^62^ and Zhu et al.^146^).

#### Participatory innovation

To determine which type of update should be performed, an important factor to consider is the effort required to collect each kind of feedback. Domain-level feedback may require focus groups and workshops.^63^ Observation-level feedback might be faster to collect (e.g., large-scale data-labeling platforms^36^), making it easy to collect dataset changes or additions.^147^ Creating efficient ways for practitioners to collect domain-level feedback may reduce the complexity of a loss function update, which might be preferable to dataset updates in resource-constrained settings.

### Conclusion

As ML is increasingly deployed in key societal settings, there is a growing need to incorporate domain expert preferences into models. Practitioners need mechanisms to gather and incorporate feedback from non-technical experts into the models they develop. In this work, we studied the interaction between practitioner and expert to see how feedback can be collected and then used to update the model itself. We propose a taxonomy to convert feedback from an expert, who can provide domain- or observation-level feedback, into model updates, which change the dataset, loss function, or parameter space. We conclude with concrete open questions that pertain to prompting for feedback, engaging with diverse feedback, and selecting the update type appropriately. We implore the community to study how to best incorporate domain expertise into the ML development cycle.
